# Repression of transcription by the glucocorticoid receptor: A parsimonious model for the genomics era

**DOI:** 10.1016/j.jbc.2021.100687

**Published:** 2021-04-21

**Authors:** Anthony N. Gerber, Robert Newton, Sarah K. Sasse

**Affiliations:** 1 Department of Medicine, National Jewish Health, Denver, Colorado, USA; 2 Department of Immunology and Genomic Medicine, National Jewish Health, Denver, Colorado, USA; 3 Department of Medicine, University of Colorado, Aurora, Colorado, USA; 4 Department of Physiology & Pharmacology and Snyder Institute for Chronic Diseases, Cumming School of Medicine, University of Calgary, Calgary, Alberta, Canada

**Keywords:** glucocorticoid receptor, inflammation, transcriptional enhancer, repression, negative feedback, GR, glucocorticoid receptor, GRE, glucocorticoid response element, nGRE, negative glucocorticoid response element, TSS, transcriptional start site

## Abstract

Glucocorticoids are potent anti-inflammatory drugs that are used to treat an extraordinary range of human disease, including COVID-19, underscoring the ongoing importance of understanding their molecular mechanisms. Early studies of GR signaling led to broad acceptance of models in which glucocorticoid receptor (GR) monomers tether repressively to inflammatory transcription factors, thus abrogating inflammatory gene expression. However, newer data challenge this core concept and present an exciting opportunity to reframe our understanding of GR signaling. Here, we present an alternate, two-part model for transcriptional repression by glucocorticoids. First, widespread GR-mediated induction of transcription results in rapid, primary repression of inflammatory gene transcription and associated enhancers through competition-based mechanisms. Second, a subset of GR-induced genes, including targets that are regulated in coordination with inflammatory transcription factors such as NF-κB, exerts secondary repressive effects on inflammatory gene expression. Within this framework, emerging data indicate that the gene set regulated through the cooperative convergence of GR and NF-κB signaling is central to the broad clinical effectiveness of glucocorticoids in terminating inflammation and promoting tissue repair.

Signaling through the glucocorticoid receptor (NR3C1, GR) is essential for normal human physiology (1, 2, 3). GR is a ubiquitously expressed nuclear hormone receptor that functions through ligand-induced translocation to the nucleus. There, GR regulates gene expression, resulting in critical physiological and pharmacological effects (see Fig. 1) that depend heavily on cellular and organismal context (4, 5, 6, 7). An example of this specificity is the indispensable role for GR signaling in lung development (8), where maturation and secretion of surfactant by cells unique to the lung require GR signaling (9, 10). Many other tissue types also perform unique responses to glucocorticoids, including the bone, nervous system, skeletal muscle, heart, and liver (11, 12, 13, 14). Circadian biology is also regulated by glucocorticoids (15). This, in collaboration with components of the core circadian clock, coordinates diverse physiological effects during health, including the regulation of metabolic and immune system homeostasis (16, 17, 18). These effects are altered by dynamic changes in corticosteroid hormone levels (19, 20), which can rewire inflammation and metabolism as an adaptive response to stress.

**Figure 1:**
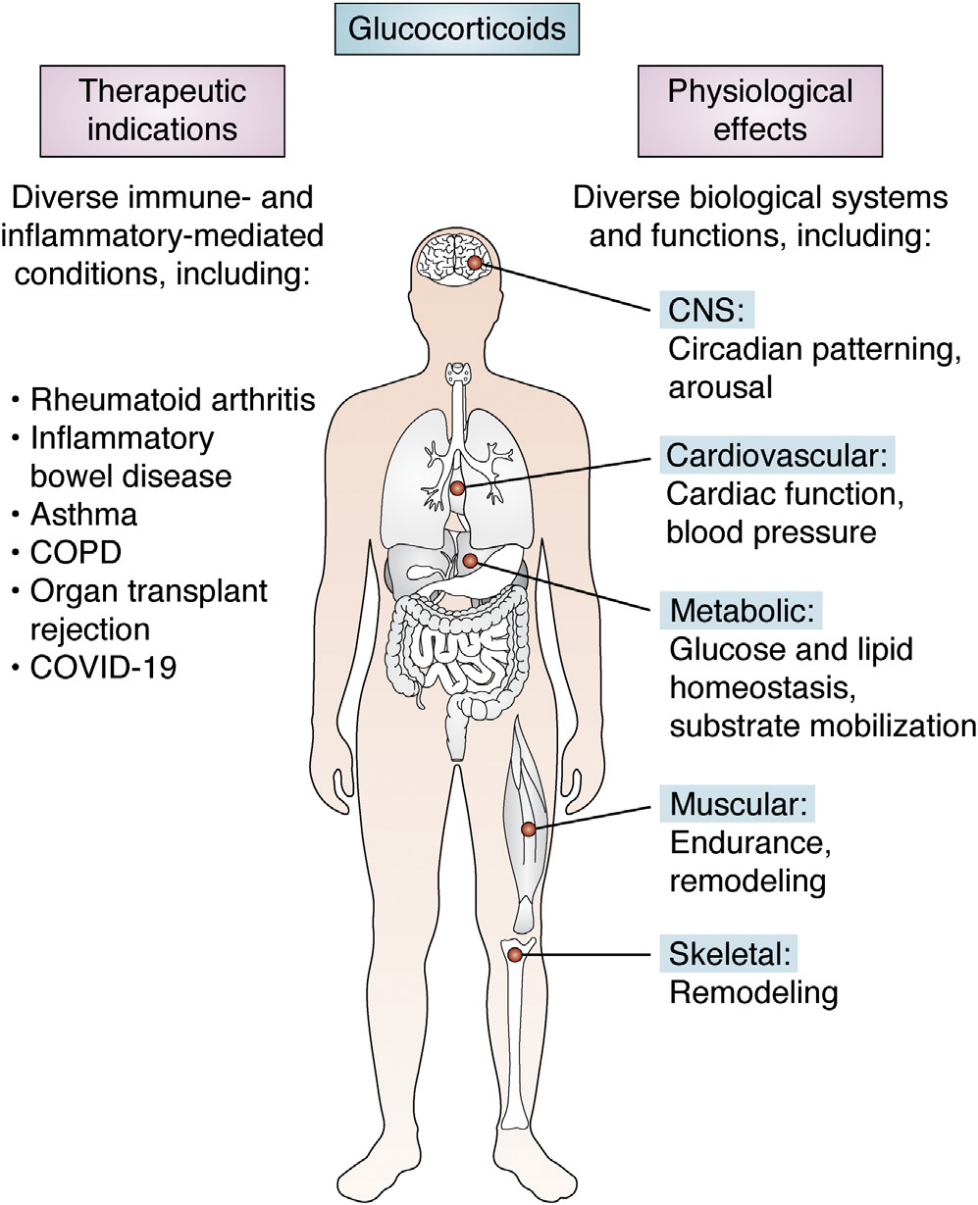
**Glucocorticoid signaling has diverse clinical indications and physiologic effects**.

Beyond these crucial roles in normal physiology (21), likely through commandeering evolutionarily favorable stress response pathways (20), it has been recognized for more than 70 years that glucocorticoids have potent anti-inflammatory properties when used as drugs (22, 23). The range of diseases that are treated with glucocorticoids is extraordinarily diverse and includes asthma, rheumatoid arthritis, spinal cord injury, vasculitis, inflammatory bowel disease, sarcoidosis, and systemic lupus erythematous, among many others (24, 25, 26, 27, 28, 29). Oral corticosteroids are also effective in treating exacerbations of chronic obstructive pulmonary disease (COPD), which are frequently caused by viral infections (30). This role in resolving viral-induced inflammation in the lung has recently been extended to treating COVID-19 disease, where moderate doses of dexamethasone administered to patients with severe COVID-19 for up to 10 days reduced mortality (31). Thus, understanding the mechanisms of GR signaling is as relevant today as it was 50 years ago, when it was first recognized that hormone-regulated changes in cellular function are driven by direct effects of ligand-activated receptor on gene expression (32, 33). Here, we propose a parsimonious model for GR-mediated transcriptional repression built on concepts validated through unbiased genome-wide investigations.

## Classical models of GR signaling

The highly inducible nature of glucocorticoid activity led to extensive use of GR signaling as a model system for pregenomics studies of eukaryotic transcription. This occurred in parallel with efforts to decipher the mechanistic basis for glucocorticoid effects on normal physiology and inflammation. Such studies, from the 1980s, 1990s, and early 2000s, defined a canonical pathway in which, following ligand binding and nuclear translocation, homodimeric GR binds to specific palindromic and near palindromic DNA sequences with high affinity (34, 35, 36, 37). Sequences with both palindromes and near palindromes, which comprise the canonical glucocorticoid response element (GRE), were found in the regulatory regions of numerous GR-induced genes, such as *TAT*, *PEPCK*, and others (35, 36, 38). Reporter analyses defined a functional requirement for these binding sites in mediating GR-driven transcription (36, 39). Characterization of functional GREs within the context of immediate flanking DNA sequences indicated that GR functions in collaboration with other transcription factors (40), in a process implicated in imparting tissue specificity to hormone responses (41). Moreover, coregulators that do not directly bind DNA, such as NCOA2 (also known as GRIP1), were shown to associate with specific surfaces of GR to enable formation of GR-nucleated multiprotein complexes (42, 43). These recruit or interact with the more general components of the transcriptional apparatus (42), including chromatin remodeling machinery (44), and ultimately RNA polymerase II (45, 46). In aggregate, these studies informed a model in which ligand-activated homodimeric GR interacts with high-affinity GREs located in regulatory regions of target genes, initiating multistep recruitment of additional proteins. This culminates in increased gene transcription in a process that depends on both the local features of the GRE and the overall cellular context (47, 48, 49). This model was largely congruent with findings for other nuclear hormone receptors and shared many features with models for the activity of tissue-specific transcription factors that drive differentiation, such as MyoD (50, 51).

**Figure 2:**
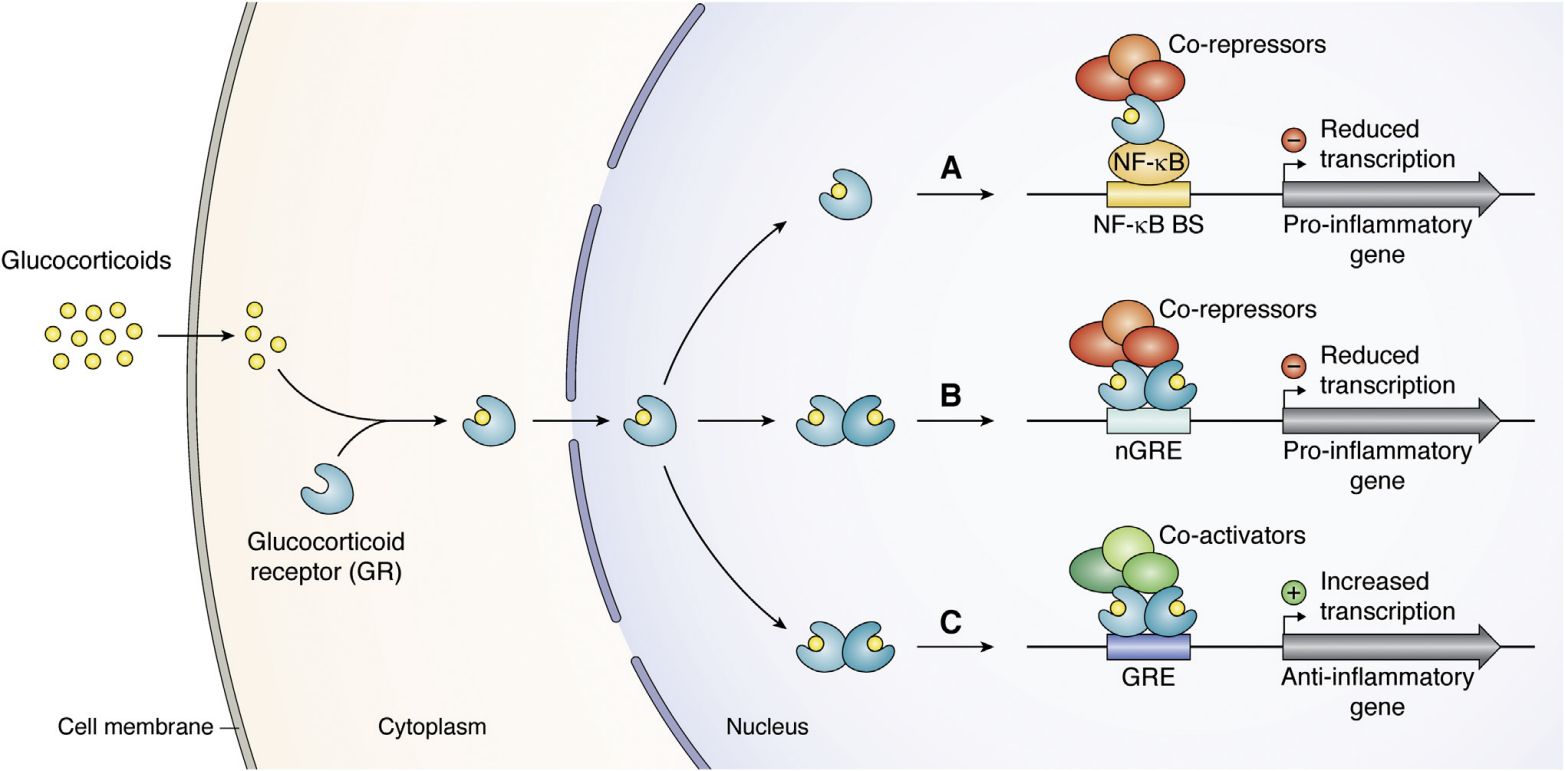
**A classical model of GR-mediated inflammatory repression.** First, cytoplasmic GR interacts with glucocorticoids, resulting in a conformational change and nuclear translocation. *A*, primary repression resulting from GR monomers tethering to inflammatory transcription factors, such as NF-κB, leading to recruitment of corepressors. *B*, GR homodimers interact with specific DNA sequences, also resulting in recruitment of corepressors and reduced transcription of inflammatory genes. *C*, secondary repression resulting from GR homodimers inducing the transcription of genes encoding proteins that repress inflammatory gene transcription, such as NFKBIA.

A number of genes induced through the canonical GR signaling pathway, *i.e.*, in association with high affinity GR:GRE interactions, were identified to have a role in the repression of inflammatory processes, *e.g.*, *TSCD22* (also known as GILZ), *NFKBIA*, and *DUSP1* (52, 53, 54, 55, 56, 57). However, repressive effects of glucocorticoids on gene expression were too rapid to be solely attributable to secondary effects of GR-induced genes, and repression did not uniformly require protein synthesis (58, 59, 60). Accordingly, numerous studies and models focused on alternate mechanisms encompassing what we will refer to as “primary” transcriptional repression, *i.e.*, not requiring protein synthesis, as contributing to the effects of glucocorticoids on repressing gene expression and inflammatory processes (Fig. 2). Some studies suggested that GR interactions with DNA through canonical or semipalindromic GREs could result in “steric” inhibition of interactions between inflammatory factors and nearby DNA elements (35, 61, 62). Other studies suggested that inductive transcriptional complexes nucleated by GR included cofactors that were recruited from other active enhancers and promoters (63). This competition consequently resulted in reduced expression of a subset of genes that are regulated by these cofactors (64, 65). Determinants of the GRE itself were also implicated (66), and GR interactions with specific DNA sequences known as negative GREs (67), which differ from canonical high-affinity GR binding sequences, were reported to result in repression rather than induction (67, 68). However, the dominant model that emerged was centered on the notion of repressive tethering or protein–protein interactions between GR and other transcription factors that resulted in reduced gene expression (69, 70, 71). In this model (see Fig. 2), without binding directly to DNA, GR associates or tethers with NF-κB or AP-1 (72, 73), resulting in repression of their activity in a process typically attributed to recruitment of transcriptional corepressors such as NCOR1 and HDAC2 (74). This model was ostensibly supported by studies of mutations that prevented GR dimerization and/or limited GR-mediated transcriptional induction. For example, largely based on reporter assays, GR dimerization mutants enabled transcriptional repression in response to glucocorticoids (72, 75). These presumptive monomeric forms of GR failed to bind DNA in biochemical assays and did not efficiently induce gene transcription through canonical GREs (72). However, GR dimerization mutants were known to support transactivation by GR in some contexts (76), thus the available data were not necessarily supportive of tethering-based repression (77). Nevertheless, canonical DNA binding and classical transcriptional induction came to be viewed as largely dispensable for GR-mediated repression (78, 79). This model formed the basis for extensive attempts to develop improved GR ligands with reduced side effects (80), none of which have resulted in clinically used drugs. As we will discuss below, although it is increasingly recognized that the bifurcated model is overly simplistic, the model continues to be incorporated in currently and serves as a conceptual framework for understanding GR signaling (81, 82, 83, 84, 85).

## Genomics studies of the glucocorticoid receptor and transcription

The development of genomics technologies afforded an opportunity to test the validity of pregenomics models of GR-mediated gene induction and repression on a genome-wide basis using entirely distinct methods. In particular, integrated studies of gene expression and interactions between GR and chromatin emerged as a powerful approach to define the characteristics and transcriptional consequences of GR interactions with specific DNA sequences. One primary theme from these studies has been robust validation of the importance of interactions between GR and the canonical palindromic and near-palindromic family of GR-binding sequences. Beginning with “ChIP-on-ChIP” assays and extending to ChIP-seq studies (86, 87), the canonical GR-binding sequence, or GRE, (GnACAnnnTGTnC), was reidentified with these new approaches. Based on proximity, interactions between GR and canonical GREs were associated with induction of transcription across the genome. Although these interactions vary based on cell type and chromatin context, a number of studies have found that ∼50% of interactions between GR and chromatin involve a palindromic or semipalindromic canonical GRE (87, 88), indicating that the sequence is central to the genomic response to glucocorticoids. In contrast, interactions between GR and negative GREs have not been reproducibly identified on a genome-wide basis (89, 90). Thus, no consensus has emerged from these genome-wide studies on the role of noncanonical DNA sequences in mediating transcriptional responses to glucocorticoids. Such interactions between GR and negative GREs, if they occur, may be limited to certain cell types or rely on specialized posttranslational modifications of GR (91).

Beyond this validation, these studies also identified unexpected features that both extended our understanding of GR signaling and raised important questions that remain under investigation. First, not all GR-binding regions could be clearly associated with transcriptional changes of nearby genes (87). Second, the chromatin structure of sites interacting with GR was variable, indicating that GR may act as a pioneer factor in some contexts, but in other contexts GR activity appears to be predicated on binding of other factors or preexisting properties of the local chromatin structure (92). Thus, it is not possible to predict GR binding to a specific genomic region based solely on its sequence. Third, and of relevance to repression, the distributions of GR interactions with chromatin relative to the transcriptional start site (TSS) of induced versus repressed genes are markedly different. For example, work by Reddy *et al* (87), found that, on average, TSSs of genes with increased expression in response to glucocorticoids are located within 11 kb of a genomic site of GR occupancy, and GR occupancy was identified near 47% of induced genes. In contrast, the average distance between TSSs of repressed genes and sites of GR occupancy was 146 kb, and GR occupancy was detected in proximity to only 8% of repressed genes. These data aligned with earlier work from So *et al* (86). Considering that regulatory elements for inflammatory genes are not uniformly distributed at great distances from TSSs (93, 94, 95), the striking difference in GR occupancy distribution suggested that the relationship between GR occupancy and transcriptional induction versus repression is fundamentally different. It is theoretically possible that tethering interactions between GR and more proximal enhancers for inflammatory genes may not be captured efficiently by cross-linking. However, ChIP-seq studies of transcriptional cofactors such as EP300, which do not bind DNA directly, have successfully defined thousands of analogous sites of indirect factor occupancy (96). Likewise, NCOR1, a corepressor that represses NF-κB regulated genes, exhibits genome-wide occupancy with significant overlap with NF-κB regulated enhancers, including enhancers in close proximity to gene bodies (97). Thus, the absence of robust and reproducible sites of GR occupancy at proximal regulatory elements for repressed genes is unlikely to be a detection artifact related to tethering interactions. Instead, these studies suggest that repression of gene expression by GR occurs in the absence of detectable tethering interactions in close proximity to repressed loci.

The absence of canonical GREs in many regions of GR occupancy observed in several studies aligns with previous data indicating that GR utilizes distinct surfaces and cofactor interactions at particular response elements (76). These interactions depend on the specific sequences within and in proximity to specific GREs. One surface of particular interest with respect to pregenomics models of repression was the GR dimerization interface. Accordingly, Schiller and colleagues performed ChIP-seq analysis comparing wild-type GR occupancy with occupancy of GR A477 T, which harbors a mutation in the dimerization interface that abrogates GR dimerization. GR A477 T exhibited widespread hormone-inducible genomic occupancy, which overlapped significantly with occupancy sites for wild-type GR (98). Although this study lacked the resolution to definitively determine whether wild-type GR occupies any of the overlapping sites as a monomer or dimer, these findings challenged the fundamental basis for pregenomics models of repressive tethering. Aligned with findings from *Nr3c1*^tm3Gsc^ mice (99), which harbor a mutation in the GR dimerization domain and other studies (76), these data instead suggest that dimerization mutations are not null with respect to DNA binding. Rather, point mutations in the dimerization domain alter the specificity of interactions between GR and chromatinized DNA within the context of the cell.

In further support of this notion, Starick *et al* used ChIP-exo, a deep sequencing-based method that combines exonuclease digestion with ChIP, to define specific DNA sequences that interact directly with GR or are bound by factors that interact with GR (100). This work identified protected GR “half sites”, *e.g.*, that lacked significant palindromic features in key residues within the flanking sequences. Moreover, in some cases these half sites were associated with protection of adjacent DNA residues that formed matches for the consensus binding site for the TEA domain (TEAD) family of transcription factors. Thus, the authors concluded that GR can interact directly with DNA as a heterodimer comprised of a GR monomer and another transcription factor, such as TEAD4. A caveat to this interpretation is that ChIP-exo cannot determine whether interactions between GR and half sites reflect homodimeric GR interactions with DNA in which only one of the two DNA-binding domains within the homodimer contacts a half site at any given time. In that regard, Hager and colleagues have shown that local on/off rates between GR and DNA are very rapid (101, 102, 103), and they have also argued that interactions between GR and canonical GREs nucleate the formation of a tetrameric form of GR that mediates higher-affinity interactions with a given GRE than a traditional homodimer (104). A similar model could extend to half sites interacting with homodimeric forms of GR. Independent of the precise mode of half site occupancy by GR, these data indicate that GR can efficiently interact with half sites, or other sequences that lack traditional contact points for the GR homodimer, and, as in the case of the A447 T mutant, without forming a homodimer. These findings largely undermine the original basis for transrepression models in which the GR monomer was reportedly unable to bind directly to DNA and activate transcription. Indeed, recent work indicates that DNA-binding activity of GR is inseparable from transcriptional repression (105), data that are at odds with the theoretical premise for repressive tethering mechanisms.

## Repression of inflammatory gene expression

To more directly explore repressive mechanisms, a number of studies analyzed molecular cross talk between glucocorticoids and inflammatory signals. These studies have demonstrated complexity in genome-wide interactions between GR and inflammatory transcription factors that belies reductionist mechanistic models for glucocorticoid-mediated inflammatory repression. For example, Rao *et al* identified six clusters of differential responses in the setting of combinatorial treatment with the GR agonist triamcinolone and TNF, which activates NF-κB, in HeLa cells (106). These clusters included genes that were coinduced by both stimuli, a finding that has also been reported by other groups and is now implicated in secondary (*i.e.*, indirect) inflammatory repression by glucocorticoids, as discussed in further detail below. Although examples of possible repressive tethering were reported by Rao *et al*, only 12% of GR-binding sites (or ∼1033/8696 sites) were evident with TNF plus triamcinolone treatment that were not detected with triamcinolone treatment alone, whereas there were over 12,000 binding sites for the p65 subunit of NF-κB identified in these experiments. A comparably modest effect of TNF on genome-wide interactions between GR and chromatin was observed in airway epithelial cells (88). Similarly, Uhlenhaut *et al*, in a comprehensive investigation of cross talk between GR, NF-κB, and AP-1, reported that only 20% of regulatory regions subject to repression by GR signaling could potentially be attributed to transrepression-based mechanisms; they also reported on eight clusters of distinct cross talk patterns between inflammatory and GR signaling (107). Thus, rather than serving as a unifying model of GR interactions with NF-κB, any occurrences of tethered GR acting in repressive mechanisms are limited to only a small proportion of NF-κB binding sites and GR-repressed regulatory regions. Moreover, these data also make clear that the genomic and sequence context of regulatory elements is crucial for determining interactions between GR and NF-κB, a complexity that was not accounted for in tethering models of repression by GR or in many of the assays used as surrogate measures of this mechanism.

To assess relationships between enhancer activity, gene transcription, and GR occupancy at a more granular level, Sasse *et al* integrated GR ChIP-seq and nascent transcript sequencing datasets from airway epithelial cells treated with TNF and/or dexamethasone (108). Aligned with observations from other groups, this work reported very rapid repression of RNA polymerase II activity within enhancers and gene bodies, in some cases within 10 min, incontrovertibly indicative of a repressive effect of GR signaling on transcription that does not rely on the actions of GR-induced genes (109). Through comparing ChIP-seq data generated with two different GR antibodies with and without GR knockdown, dexamethasone-mediated induction of GR signaling resulted in rapid repression of the activity of some regulatory elements, as defined by nascent transcription signatures, without any detectable local GR occupancy at many of the repressed genomic sites. Sasse *et al* also noted that the activity of many regulatory regions subject to NF-κB-mediated induction was repressed by GR signaling in the absence of TNF, similar to prior observations (109). This repression was associated with rapid local chromatin remodeling and further indicates that GR interactions with NF-κB are not required for repression of inflammatory targets of TNF signaling by glucocorticoids. Moreover, these changes in chromatin structure were observed to influence the specificity and efficiency of ChIP-based assays at these regulatory regions, which potentially complicates accurate quantification of factor occupancy at these sites.

**Figure 3:**
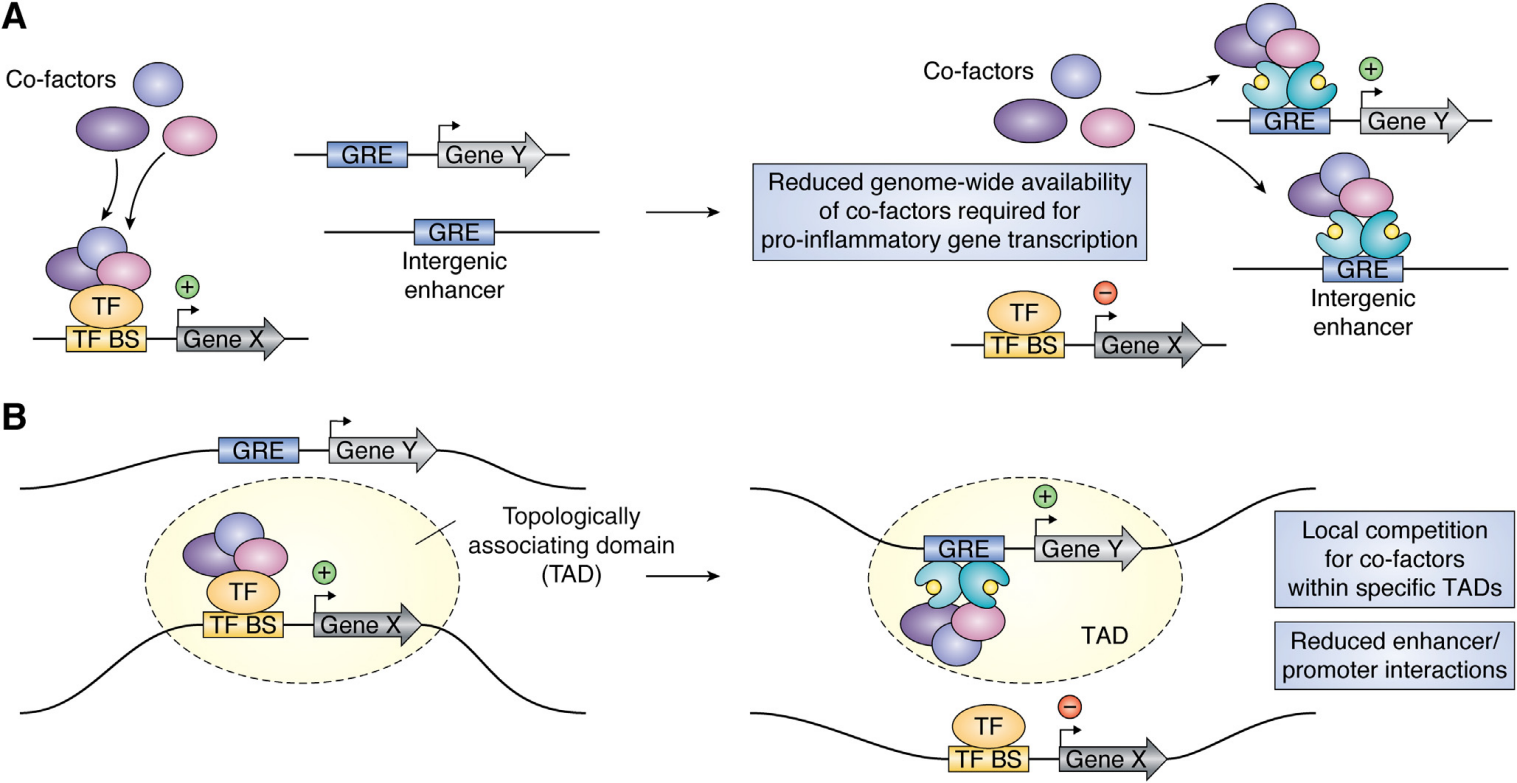
**Two models of competition-based transcriptional repression by GR.***A*, in a standard “squelching” model of competition, GR shares limiting quantities of cofactors with other transcription factors (TF) across the nucleus. Consequently, recruitment of cofactors to GR:GRE complexes results in reduced availability of coregulators for other enhancers, resulting in repression. *B*, in this “local competition” model, rather than competition throughout the nucleus, specific transcriptionally productive GR:GRE interactions reduce the activity of subsets of enhancers within shared topologically associated domains (TAD). This could include local competition for required cofactors and a loss of enhancer–promoter interactions in three dimensions. A GR:GRE interaction that is associated with induction of a nearby gene is depicted; however, isolated GR:GRE interactions that result in enhancer RNA transcription not clearly linked to gene transcription could also result in local competition with other enhancers.

Considering the data currently available, we contend that standard formulations of transrepression mediated by negative GREs or tethering, which continue to enjoy broad acceptance in the literature, should no longer comprise the starting point for understanding glucocorticoid-mediated transcriptional repression. Instead, mounting evidence indicates that some form of competition generally underlies primary repressive effects by GR (110, 111). What do available data tell us about competitive inhibition of transcription associated with canonical GR:GRE interactions in the genomic context? At least two nonexclusive possibilities provide potential mechanistic explanations (Fig. 3). First, it has been proposed that repression is a consequence of squelching (112), a form of competition in which certain transcriptional regulators are in limited quantities within the nucleus (64, 113). In traditional squelching models, these regulators would be titrated on a genome-wide basis from their original sites of activity to new transcriptional complexes nucleated by GR at GRE-centered regulatory regions. In support of this mechanism, decreases in EP300 occupancy at repressed regions in association with increased EP300 occupancy at GR-induced regions have been reported (111). The temporal pattern of reduced signals of EP300 occupancy at repressed regulatory regions, along with weak and transient GR interactions with some of these regions, was used to argue for factor redistribution in a squelching-type mechanism (111). A similar model for repression of gene expression by the estrogen receptor has also been proposed (113). As noted above, however, changes in local chromatin structure can influence quantification of factor occupancy in ChIP-seq assays, potentially complicating quantitative conclusions regarding factor redistribution. Moreover, based on changes in enhancer RNA transcription (108), glucocorticoids repress the activity of only a small set of enhancers relative to genome-wide occupancy of EP300 and the well-established global role EP300 plays in metazoan transcription (96). Thus, traditional squelching models are not entirely consistent with available data on the primary repressive effects of GR on transcription.

In a further challenge to genome-wide competition models, growing evidence indicates that transcription frequently takes place in localized areas within the nucleus, often referred to as transcription factories (114). Three-dimensional relationships between different enhancers and TSSs that comprise individual transcription factories are studied using chromatin conformation capture techniques, and specific areas of active transcription within the nucleus can be visualized microscopically as phase-separated condensates (115). The structural properties and local concentrations of transcription factors and other components of the transcriptional machinery within condensates differ significantly from other regions within the nucleus (116, 117). Thus, GR-induced and repressed regulatory regions with shared nuclear positioning would be predicted to be in local competition for common cofactors within condensates or nuclear subdomains, as suggested for other regulatory factors and domains (118, 119, 120, 121). This process could assign biologic activity to GR:GRE interactions that are not clearly linked to changes in gene transcription in linear genomic space, yet increase transcription of enhancer RNAs, whose direct function in transcriptional regulation is not yet fully defined. Moreover, weak interactions between GR and chromatin (122), which have been variably reported at repressed regulatory regions (111), could result from close three-dimensional nuclear proximity of repressed regions with sites of canonical GR:GRE interactions. Irrespective of the underlying mechanism, as reviewed by Schmidt *et al* (112), numerous signaling cascades (*e.g.*, glucocorticoid signaling, estrogen signaling, TNF) that activate specific transcription factors cause both inductive and repressive effects on transcription (108, 123, 124). Rather than highly specialized repressive interactions occurring for a wide range of transcription factors, many of which lack classical repressive domains, a competition model provides a simple and unified explanation for this property of signal-activated transcription factors.

**Figure 4:**
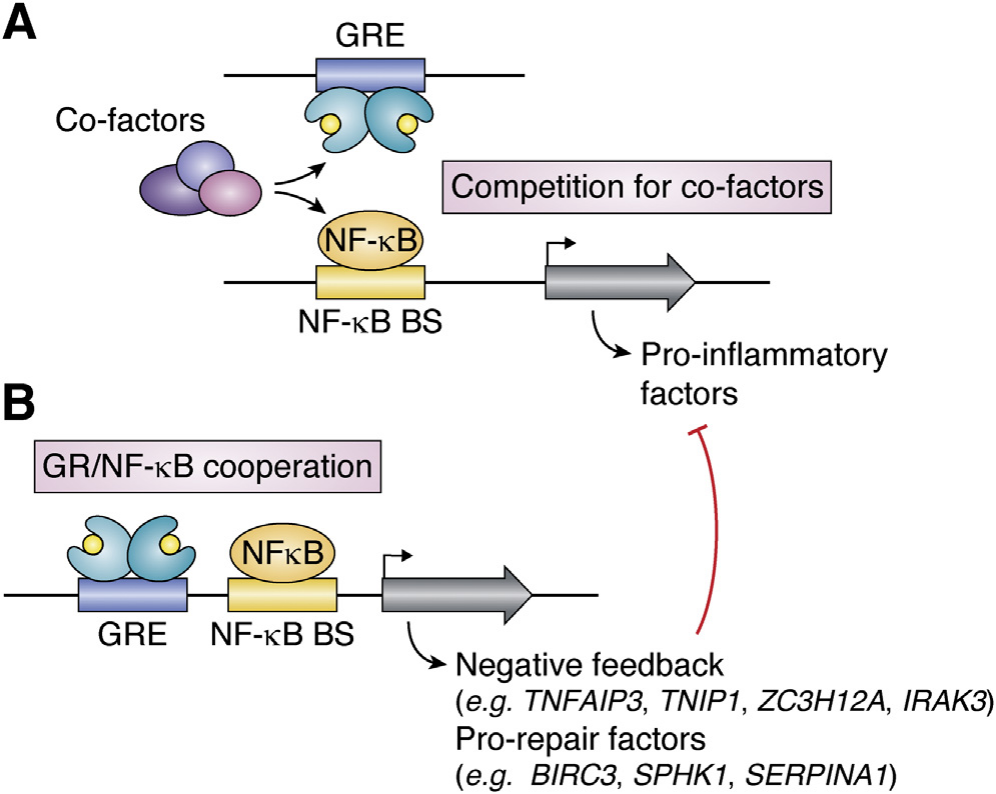
**A parsimonious two-part model for repression of transcription and inflammation by the glucocorticoid receptor through cross talk with NF-κB.***A*, first, activation of GR signaling causes rapid, primary repression of NF-κB through competition for cofactors between canonical GR:GRE complexes and a subset of NF-κB-regulated enhancers. Whether competition for factors is nucleus-wide or occurs through spatially restricted relationships between GRE and NF-κB motifs remains to be determined. *B*, in addition to primary repression due to competition, enhancers with GR and NF-κB binding motifs nucleate transcriptional cooperation between these two factors. GR cooperation with NF-κB exerts secondary repression through augmenting the expression of negative feedback regulators of inflammation and NF-κB (*e.g.*, *TNFAIP3*, *TNIP1* (142), *ZC3H12A* (143), *IRAK3*) and factors that promote inflammatory resolution and repair (*e.g.*, *BIRC3*, *SPHK1*, *SERPINA1*). Although not depicted, additional induced targets of GR that appear to be regulated without NF-κB cooperation, such as *TSC22D3*, also contribute to secondary repression.

If it is a general property that programs of strongly induced transcription necessarily cause reciprocal repression (112), how does this relate to the uniquely broad and efficacious anti-inflammatory effects of GR signaling? We speculate that underlying these potent effects is a unique spatial and evolutionary relationship between functional GR and NF-κB motifs consistent with a simplified two-step model we propose for inflammatory repression (Fig. 4). For primary transcriptional repression in this model, the nuclear positions of specific GR-occupied GREs are in close three-dimensional proximity with subsets of NF-κB-regulated enhancers that are subject to primary transcriptional repression by glucocorticoids. Whereas this facet of our model remains to be definitively established, it is known that a significant subset of enhancers with NF-κB/p65 occupancy are strongly enriched for canonical GREs with much higher predicted affinity for GR than would be expected to occur randomly (88, 106). Many of the enhancers harboring motifs for both factors are associated with cooperation between GR and NF-κB, indicative of evolutionary pressure for this regulatory paradigm, which we propose as central to secondary repression. Genes regulated through GR-NF-κB transcriptional cooperation include a broad range of anti-inflammatory and pro-repair genes, with a growing body of evidence indicating that induction of negative feedback is indispensable for inflammatory repression by glucocorticoids (125). For example, *Irak3* is regulated cooperatively by GR and NF-κB and is required for beneficial effects of glucocorticoids in a mouse model of bacterial infection (126). Similarly, *Sphk1*, which is cooperatively induced by glucocorticoids and inflammatory signals in macrophages, is essential for a glucocorticoid-mediated repression of acute lung injury in response to lipopolysaccharide (127). *TNFAIP3* (A20), a primary negative feedback regulator of NF-κB implicated in GR-mediated repression of inflammatory gene expression, is regulated in part through potent cooperation between GR and NF-κB within an intronic enhancer. Even *DUSP1*, often reported as a GR-induced gene that represses inflammation (54, 128, 129), shows maximal induction in response to combined GR activation and inflammatory signals (130), and analysis of published ChIP-seq and nascent transcript sequencing data defines a potential cooperative enhancer ∼80kb upstream from the *DUSP1* TSS (108). Considered together, the specific enhancer set subject to primary repression in response to GR signaling, coupled with GR-NF-κB cooperatively augmenting the expression of key negative feedback, or feedforward, regulators of inflammation and pro-repair genes comprise a combination that appears unique to GR signaling. These two activities thus provide a framework to understand the remarkably effective anti-inflammatory properties of glucocorticoids.

## Future directions

This revised model of the mechanistic basis for the effects of GR signaling on the repression of inflammatory gene transcription affords numerous opportunities for future research. Genomic editing of GREs, which is now feasible with improved editing methodology (131), guided by deep conservation analysis and three-dimensional maps of chromatin organization, could be applied to investigate the mechanistic basis for primary transcriptional repression. Consider the case where primary repression of specific inflammatory gene enhancers results from local competition with a small corresponding group of inductive GR:GRE complexes (*i.e.*, GR:GRE interactions that induce local enhancer RNA transcription). In this scenario, editing selected GREs would abrogate primary repression for a subset of inflammatory genes in a “jackpot” effect. A jackpot-type effect in which disrupting a small set of GREs impacts repression on a corresponding small set of enhancers would support spatially constrained “local” competition as an explanation for primary repression by GR (Fig. 3*B*). Such an effect, however, would not disprove a role for more traditional genome-wide squelching (Fig. 3*A*), nor would it definitively refute tethering or nGRE mechanisms contributing to repression at some loci. In that regard, current models for inflammatory repression by GR are frequently presented as “add-ons” to older models based on experimental approaches now recognized as having clear limitations (24). The inherent difficulty in disproving a possible molecular interaction, such as repressive tethering, should not be a requirement for accepting new models that shift focus away from older concepts, some of which have yet to be definitively established with powerful contemporary techniques.

Beyond the mechanistic basis for primary repression, our understanding of the clinical effectiveness of glucocorticoid signaling continues to evolve and suggests important new avenues for research. For example, the model presented above is largely based on studies with NF-κB, which is of therapeutic relevance in many diseases treated with glucocorticoids, such as rheumatoid arthritis and inflammatory bowel disease (27, 132). However, glucocorticoids are also used in millions of patients to treat Type 2 inflammation, which is generally a consequence of the activity of other transcription factors, notably STAT6 (133). Surprisingly, comparatively little is known at the molecular level about cross talk between GR and the STAT family, although a genome-wide study of GR cross talk with STAT3 suggested complexity in cross talk patterns similar to studies on GR and NF-κB (89). It is tempting to speculate that our model for repression of NF-κB-driven inflammation by GR could serve as a framework to understand how glucocorticoids inhibit Type 2 inflammation. Specifically, in the appropriate cell type, “Type 2” inflammatory enhancers might be poised for primary repression by induction of GR signaling based on nuclear proximity and/or shared coregulators as described above for primary repression of NF-κB targets. Cooperation between GR and Type 2 inflammatory transcription factors, such as STAT6, could also serve to augment negative feedback control of Type 2 inflammation, possibly through regulation of genes such as *SOCS1* and *PTPN1* (134, 135). Additional genome-wide studies are needed to explore the basis for GC efficacy in Type 2 inflammation and the role of negative feedback control in mediating repression of Type 2 inflammation. In that regard, analysis of steady-state RNA levels can miss cooperative control of genes that are regulated combinatorially by noncooperative and cooperative enhancers (88, 108), suggesting that higher-resolution approaches may be needed to identify potential cooperation between GR and other transcription factors.

In addition to treating Type 2 inflammation in asthma, glucocorticoids have been used for decades to treat the consequences of viral infections in asthma, and they have a similar role in treating exacerbations of chronic obstructive pulmonary disease, which are also typically driven by viral infections. Most recently, glucocorticoids have emerged from a crowded field of newer therapies, such as IL-6 blockade, to become the anti-inflammatory treatment of choice for COVID-19 disease (31). Although little is known about the anti-inflammatory effects that glucocorticoids exert directly on Type II alveolar epithelial cells, a primary target of SARS-CoV-2 infection, in airway epithelial cells, cooperation between GR and NF-κB regulates a wide range of genes with clear relevance to inflammatory repression and lung repair (88, 108). These include *TNFAIP3 (A20)*, a ubiquitin editor that represses NF-κB activity (136, 137), and *SERPINA1*, which encodes alpha1 anti-trypsin and is implicated in preventing SARS-CoV-2 cellular entry (138, 139). Transcription of *BIRC3*, which inhibits apoptotic responses to inflammation in influenza infection (140), is also cooperatively regulated by GR and NF-κB. Moreover, levels of TNF, a potent inducer of NF-κB, are elevated in COVID-19 disease (141), suggesting that the cooperative mechanism may be an important component of the success of glucocorticoid-based therapies in this disease. However, the role of this cooperative activity in treating COVID-19 remains speculative. Indeed, despite decades of research and use in the clinic, many facets of glucocorticoid use continue to be based on empiric discovery rather than deriving from molecular understanding. The model for repression of inflammatory gene expression by GR that we present here provides a refreshed context for future research on this versatile signal-activated transcription factor.

## Conflict of interest

The authors declare that they have no conflicts of interest with the contents of this article.
